# Electrovectorcardiographic study of left ventricular aneurysm in ischemic heart disease

**DOI:** 10.3389/fcvm.2023.1275194

**Published:** 2023-12-12

**Authors:** Leonardo Paschoal Camacho Varoni, Nelson Samesima, Mirella Facin, Horácio Gomes Pereira Filho, Bruna Affonso Madaloso, Wilson Mathias Junior, Carlos Alberto Pastore

**Affiliations:** ^1^ Clinical Unit of Electrocardiography, Instituto do Coracao (InCor), Hospital das Clínicas FMUSP, Faculdade de Medicina, Universidade de São Paulo, São Paulo, Brazil; ^2^ Echocardiography Unit, Instituto do Coracao (InCor), Hospital das Clínicas FMUSP, Faculdade de Medicina, Universidade de São Paulo, São Paulo, Brazil

**Keywords:** heart disease, electrocardiography, echocardiography, magnetic resonance imaging, cardiac imaging techniques, myocardial infarction, coronary artery disease, left ventricular ejection fraction

## Abstract

The aim was to characterize the electrovectorcardiographic pattern of ventricular aneurysms in ischemic cardiopathy by analyzing the cardiac ventricular repolarization. The medical records of 2,670 individuals were analyzed in this cross-sectional study. A test phase included 33 patients who underwent transthoracic echocardiogram with ultrasonic enhancing agent, electrocardiogram, and vectorcardiogram (aneurysm group - *n* = 22, and akinesia group - *n* = 11). In the validation phase, cardiac magnetic resonance imaging established the left ventricle segmental contractility in 16 patients who underwent electrocardiographic and vectorcardiographic tests (aneurysm group, *n* = 8, and akinesia group, *n* = 8). The variables studied were the presence of the T-wave plus-minus pattern and the T-wave loop anterior-posterior pattern in V2–V4. The diagnostic indices used were sensitivity, specificity, and predictive values, with their respective 95% confidence intervals. During the test and validation phases, the analysis of the presence of the T-wave plus-minus pattern identified the aneurysm group with a sensitivity of 91% vs. 87% and specificity of 91% vs. 87% (*p* < 0.0001 vs. *p* = 0.01), respectively. Meanwhile, the T-wave loop anterior-posterior pattern evidenced sensitivity of 95% vs. 77% and specificity of 91% vs. 87% (*p* < 0.0001 vs. *p* = 0.04), respectively. The electrovectorcardiographic parameters showed high accuracy for recognizing left ventricular aneurysms in ischemic heart disease.

## 1. Introduction

Ventricular aneurysms occur in up to 5% of cases after acute myocardial infarction (1) and are caused by transmural anterior wall infarctions (2). Considered a late complication of myocardial infarction (after 6–8 weeks) (3), their main characteristic is to be regions that present dyskinetic movements during ventricular systole (4). Their presence confers mortality up to 6 times higher (5), with the main clinical complications being heart failure, thromboembolic events, angina and symptomatic ventricular arrhythmias (1, 2, 4–6).

Left ventricular aneurysm is characterized by presenting focal dilatation and thinning (remodeling) with either akinetic or dyskinetic systolic deformation (7). Nowadays, diastolic distortion has gained strength, even when there is no evidence of an anatomic aneurysm (large neck) (8). In addition, left ventricular aneurysm is a distinct portion of the myocardial composed of abnormal diastolic relaxation and dyskinetic paradoxical systolic movement (9).

The evolution of reperfusion therapies and the treatment of chronic heart failure led to changes in the presentation of aneurysms (10), which can be volumetrically more negligible or even non-existent depending on access to the full range of therapy. While no strong-resolution diagnostic methods were available in the past for diagnosing tiny dyskinetic portions (11), their expressions of their existence may be limited in the present time (12).

In the last 70 years, many authors have studied the forms of expression of ventricular aneurysms are expressed from an electrocardiographic point of view in patients with ischemic heart disease (13–17). However, the results present a severe limitation for a broad and efficacious clinical application for recognizing individuals with a ventricular aneurysm.

Thus, the objective of the present study aims to recognize patients with chronic ischemic heart disease and ventricular aneurysms, using well-defined criteria forwarded by the electrocardiogram (ECG) and vectorcardiogram (VCG).

## 2. Materials and methods

### 2.1. Ethical issues

The ethics committee of Hospital das Clinicas da Faculdade de Medicina da USP (HCFMUSP), São Paulo, Brazil, approved all experimental protocols of this cross-sectional study (CAAE 11678919.8.0000.0068) by Approval #5.283.660. All methods were carried out in accordance with relevant guidelines and regulations for diagnostic methods.

All the patients were recruited to the study after signing a written informed consent to have their medical records searched for data collection; additionally, if they fit the inclusion criteria, they agreed to participate in the research's test and validation phases. The study findings will help future patients who may present with the characteristics leading to the suspicion of a ventricular aneurysm and potential complications.

To protect participants’ data and privacy, data were anonymized soon after the collection.

### 2.2. Study design

Cross-sectional, observational, comparative study between two groups of patients, with and without ventricular aneurysm, designed in two stages, test and validation. This is a single-center study, impacted by the difficulty of recruiting patients during the pandemic and the cost of acquiring echocardiographic contrast (SonoVue®), which was limited to 35 units. In the test phase, the primary goal was to characterize the electrovectorcardiographic pattern of the left ventricular aneurysm in ischemic heart disease, while the secondary objective was to associate the electrovectorcardiographic findings with the left ventricular ejection fraction. Subsequently, the information gathered in the test phase was applied to those in the validation phase to verify if the results would be the same for this new population. Furthermore, the analysis of the reproducibility and agreement of the electrovectorcardiographic findings were added to the validation phase.

Inclusion criteria enrolled patients consecutively with a diagnosis of myocardial infarction (18) (rise and/or fall of cardiac troponin values with at least one value above the 99th percentile upper reference limit) with occlusion of the anterior descending artery, who, prior to the ECG, in both test and validation phases presented Q wave myocardial infarction; then, either echocardiography or cardiac magnetic resonance imaging investigated alterations in segmental contractility (dyskinesia and/or akinesia).

The following findings excluded the patient from the study: non-sinus rhythms (atrial fibrillation, atrial flutter, and atrial tachycardia), left bundle-branch block, cardiac ventricular artificial pacing, cardiac aneurysm located in the basal or middle segments of the inferior or lateral walls, cardiac aneurysm of non-ischemic cause and the presence of known hypersensitivity to sulfur hexafluoride. The presence of left and right fascicular blocks, as well as the left ventricular hypertrophy did not obstruct the electrovectorcardiographic analyses.

The database from the Technical Coordination of Diagnostic Imaging of the institution where the work was carried out provided the list of patients.

The data on which human subjects' were collected ranged between August 2019 and May 2022. The study was conducted between May 2019 and April 2023.

In the test phase, the patient's cardiac magnetic resonance imaging records performed between 2012 and 2018 were selected. Therefore, to be included in the test phase, previous CMR presenting segmental contractility abnormality was a primary condition. As the segmental contractility might change through the years, after patient selection, they underwent transthoracic echocardiography examination with an ultrasonic enhancing agent (SonoVue®) (12, 19, 20). The allocation of a patient in one of the two groups, Aneurysm or Akinesia, was defined by the echocardiographic finding. All patients underwent an ECG and a VCG. The study researcher performed all three exams (echocardiogram, ECG, and VCG) at the same day. Confirmation of the ECG reports was performed by a different cardiologist, while two other cardiologists conducted the electrovectorcardiographic analysis. In case of disagreement, a third cardiologist was consulted. The electrovectorcardiographic analysis took place later, with the analyst blinded to the patients' information.

In the validation phase, CMR imaging records performed in 2021 were selected. To define segmental contractility in this phase we selected patients who had a recent CMR examination (less than one year on average from the ECG), which waived the need to perform a new functional study (echocardiogram with an ultrasonic enhancing agent). All patients underwent ECG and VCG tests at the same day.

The electrovectorcardiographic findings from the test phase were applied to new patients included in the confirmation phase in order to verify the results obtained initially.

Additionally, the validation phase included a concordance study involving two cardiologists with different VCG reading skills. First, we analyzed the electrovectorcardiographic reading agreement between the principal examiner and a Reviewer-1, who was familiar with the methods used (30 years of experience in reading VCGs) and blinded to which groups the patients belonged. Previous training was provided to Reviewer-1 to allow recognition of the plus-minus pattern on the ECG and the anterior-posterior morphology on the VCG. Then, also regarding the vectorcardiographic findings concordance study, an additional analysis checked the agreement between the principal examiner and a Reviewer-2, who was less familiar with the VCG (three years' experience), blinded to the patients' groups and trained to recognize the proposed patterns.

### 2.3. Definitions and outcomes

#### 2.3.1. Transthoracic echocardiogram

Ultrasonic digital devices Vivid E954 (General Electric, Milwaukee, Wisconsin, USA) and Philips Medical System IE33 (Phillips, Andover, Massachusetts, USA) were used to perform these tests. The ultrasonic enhancing agent was SonoVue® (Bracco Medical Imaging, Bothel, Washington, USA).

#### 2.3.2. Cardiac magnetic resonance imaging

These exams were retrospectively reviewed and analyzed, aiming to screen patients for the test phase, and establish the segmental contractility of the left ventricle for the confirmation phase.

#### 2.3.3. Resting electrocardiogram

12-lead resting ECG Mortara Eli 250C (Mortara Co., Milwaukee, Wisconsin, USA) and TEB C10 + C30 + VS1 RV (TEB Tecnologia Eletrônica Brasileira, São Paulo, Brazil) devices were used for these tests with rhythm strip in DII with 25 mm/s paper speed and 10 mV gain (21).

The ECG analysis focused on the six leads of the transverse plane (V1–V6) for the detection of the T-wave plus-minus pattern.

Classification of the T-wave was defined from the PQ baseline (end of the PR segment and beginning of the QRS complex). The plus-minus pattern was set when the T-wave began above the baseline and ended beneath it (negative deflection).

#### 2.3.4. Vectorcardiogram

A Fukuda Denshi model HPM 7100 BSPM (Fukuda Denshi Inc., Tokyo, Japan) and a model TEB C10 + C30 + VS1 RV (TEB – Tecnologia Eletrônica Brasileira, São Paulo, Brazil) equipment were used to perform these tests. The Frank method (22) was used in both devices to obtain the three planes (sagittal, transverse, and frontal).

The gain of vectorcardiographic loops was initially acquired with a sensitivity of 4 cm/mV (sensitivity = 4). If the tracings presented overlapping loops or difficulty in identifying the P- and T-wave loops, a sensitivity of 8 cm/mV was used (sensitivity = 8). Sensitivity does not influence the angle, only the amplitude of the loops (23). This study analyzed the corrected orthogonal derivation of the transversal plane.

The VCG analysis initially also focused on the six leads of the transversal plane (V1–V6) for the detection of the anterior-posterior pattern of the T-loop. However, after the initial data collection, we chose to restrict the electrovectorcardiographic analysis to leads V2–V4.

The definition of the anterior-posterior pattern followed the convention used for vectorcardiographic analysis, which establishes the anterior and posterior location of the recorded electrical phenomena, as described below:

In the vectorcardiographic analysis of the transversal plane, we have V2 located at +90°, while V3 is at +75° and V4 at +60°, approximately. The T-loop in V2 is located posteriorly between angles 0° and −180°. Concerning lead V3 (+75°), the T-loop is located posteriorly between the angles −15° and +165°. Finally, V4 (+60°) has a posterior T-loop between the angles −30° and +150°. In other words, the T-loop is anterior in V2 and/or V3 and/or V4 between 0° and +150° (24).

**Figure 1:**
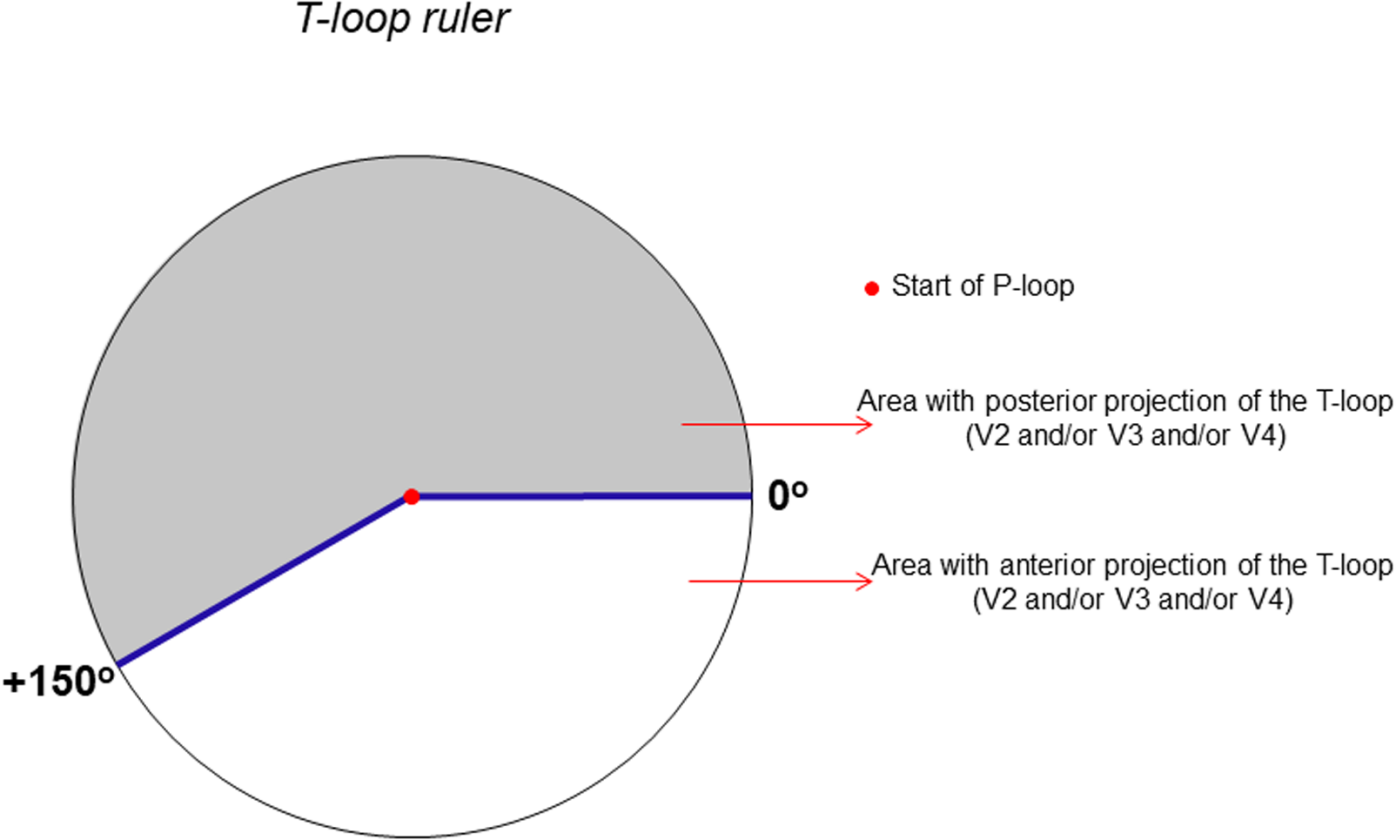
T-loop ruler: analysis of the anterior-posterior pattern of the T-loop in V2 and/or V3 and/or V4.

To facilitate the recognition of the anterior-posterior pattern, we created a T-loop ruler (Figure 1) that allowed the determination of which type is the T-loop. To do so, the professional must position the center of the T-loop ruler (red dot) on the beginning of the P-loop in the transverse plane. If the T-loop appears in the white and gray areas and starts within the white area, we see the anterior-posterior pattern.

**Figure 2:**
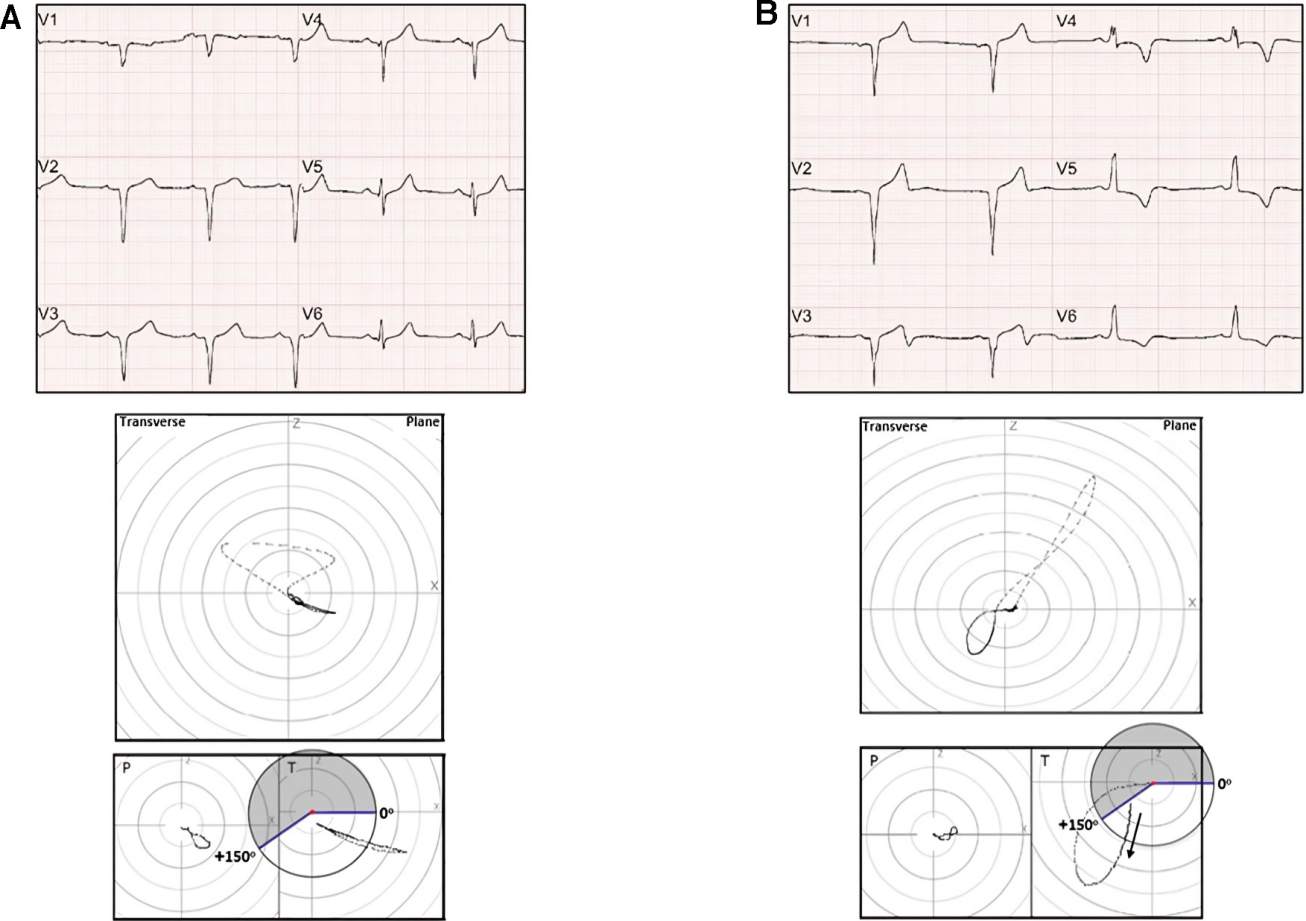
(**A**) Upper panel: ECG – anterior Q wave MI (with positive T-waves). Intermediate panel: VCG (transverse plane) – anterior myocardial infarction area. Lower panel: T-loop ruler – anterior T-loop. (**B**) Upper panel: ECG – anterior Q wave MI (T-wave *plus-minus* pattern in V3). Intermediate panel: VCG (transverse plane) – anterior myocardial infarction area and T alterations. Lower panel: T-loop ruler, T-loop with anterior-posterior morphology.

**Figure 3:**
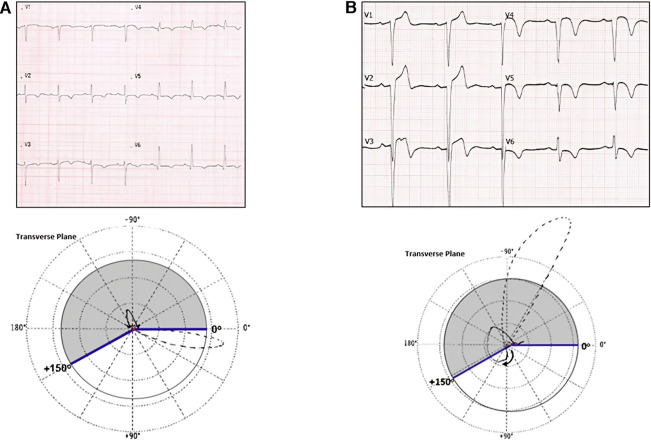
(**A**) Upper panel: ECG – suggestive of anterolateral Q wave MI (with negative T-waves). Lower panel: VCG (transverse plane) – anterolateral myocardial infarction area and T alterations. T-loop ruler, posterior T-loop. (**B**) Upper panel: ECG – anterior Q wave MI (T-wave *plus-minus* pattern in V2–V4). Bottom panel: VCG (transverse plane) – anterior myocardial infarction area and T alterations T-loop ruler – T-loop with anterior-posterior morphology.

Figures 2,3 show examples of electrovectorcardiographic analyses performed for the classification of the T-wave and T-loop on the ECG and VCG, respectively. The arrow indicates the beginning of the T-loop.

### 2.4. Statistical analysis

Quantitative clinical variables were described as mean and standard deviation, and qualitative variables were presented as absolute and relative values, after normality was assessed using the Kolmogorov-Smirnov test.

In the test and validation phases, diagnostic measures of sensitivity, specificity, positive predictive value and negative predictive value were calculated with the respective confidence intervals (95%). In this way, the researchers evaluated the discriminatory capacity of the tests (ECG and VCG) in the identification of patients with ventricular aneurysms, as well as the discriminatory capacity of the T-wave plus-minus pattern and the T-loop anterior-posterior pattern for identification of the patient with LVEF ≤ 40%.

The relationship between the T-wave plus-minus pattern and the left ventricular ejection fraction, as well as the association between the T-loop anterior-posterior pattern and the left ventricular ejection fraction were assessed using the mean and standard deviation, and compared using the *t*-test for non-equal variances, which was determined after performing the Levene test.

In the validation phase, the reproducibility in identifying the T wave plus-minus pattern (ECG) was tested with the results obtained from reviewer-1. For the reproducibility test for detecting anterior-posterior T-loop morphology (VCG), the results of reviewer-1 and reviewer-2 were analyzed. In both methods (ECG and VCG), Cohen's Kappa coefficient (*κ*) was used.

Our study used a convenience sampling method to select the participants. This approach allowed us to gather data from the participants who underwent echocardiographic contrast. We had a significant limitation due to the amount of echo contrast (thirty five units). It is important to point out that we did not find any such resources that have studied this subject, which is also why we used a convenience sampling method.

The analyses were performed using the IBM-SPSS for Windows version 20.0 software and tabulated using the Microsoft-Excel 2003 software, with a significance level of 5%.

### 2.5. Patient and public involvement in the investigation

Patients and/or the public were not involved in the design, or conduct, or reporting, or dissemination plans of this research.

## 3. Results

In the test phase, from a list of 894 patients, 142 were screened. The patients' exclusion was due to the impossibility of peripheral venous puncture, the presence of artificial cardiac pacing, and acute hypertensive edema (*n* = 3). Of the 139 remaining participants, we performed 35 examinations due to the limited accessibility and acquisition of echocardiographic enhancing agents. After analysis of the exams, two patients were excluded due to the absence of an anterior Q wave MI, and the presence of an aneurysm only in the inferior wall.

**Figure 4:**
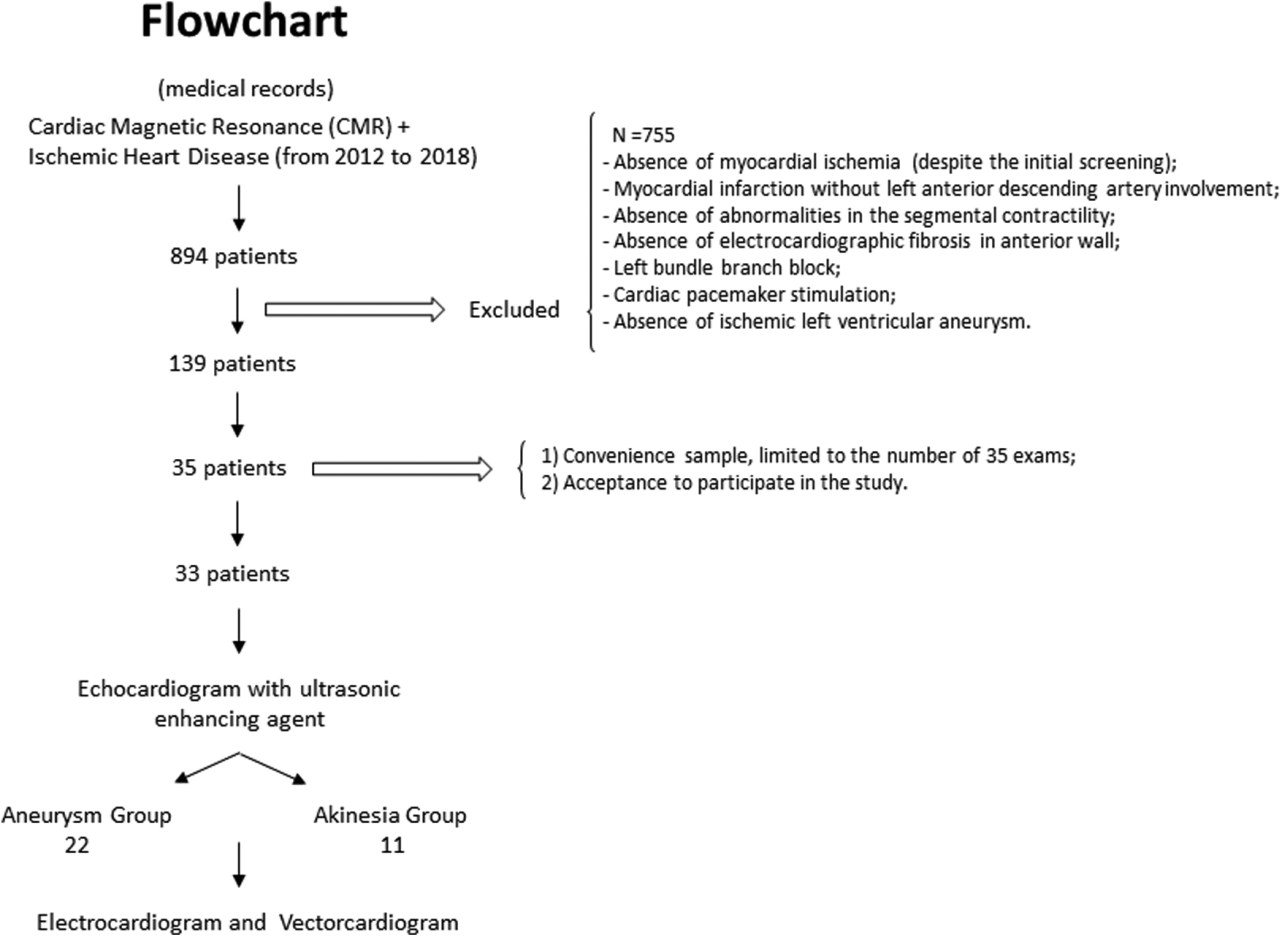
Flowchart of the participants’ selection in the test phase.

Overall, 33 patients were included and were allocated into two groups: aneurysm group (*n* = 22) and akinesia group (*n* = 11) (Figure 4).

The study population characteristics showed that the mean age in the aneurysm group was 59.4 ± 8.0 years, while in the akinesia group it was 59.7 ± 8.4 years (*p* = 0.9), with a predominance of men in both groups (77.2% in the aneurysm group vs. 81.8% in the akinesia group, *p* = 1.0). In addition, the time between acute coronary syndrome and the exams (ECG and VCG) was 55.9 ± 45.1 months in the aneurysm group and 44.6 ± 35.0 months in the akinesia group (*p* = 0.4).

The analysis and classification of the T-wave in the precordial leads, assessed by ECG, showed the presence of the T-wave plus-minus pattern in V2 and/or V3 and/or V4, in the aneurysm group, with sensitivity = 91%; specificity = 91%; positive predictive value = 95%; negative predictive value = 83%; accuracy = 91%, and likelihood ratio = 10 (*p* < 0.0001). VCG analysis in the transverse plane identified the T-loop anterior-posterior pattern in V2 and/or V3 and/or V4, in the aneurysm group, with sensitivity = 95%; specificity = 91%; positive predictive value = 95%; negative predictive value = 91%; accuracy = 94%, and likelihood ratio = 10; *p* < 0.0001 (Table 1 and Supplementary Table S1, Panels A,B).

**Table 1:** Aneurysm group identification, according to the presence of *plus-minus* (T-wave)/anterior-posterior (T-loop) in the **test phase** (segmental contractility analyzed by transthoracic echocardiography with an ultrasonic enhancing agent).

	Group		Total (*N* = 33)	*p*-value	Sensitivity	Specificity	PPV	NPV
	Aneurysm (*N* = 22)	Akinesia (*N* = 11)			(CI 95%)	(CI 95%)	(CI 95%)	(CI 95%)
Electrocardiogram (*Plus-Minus*)				**<** **.** **0001**	91%	91%	95%	83%
Yes	20 (61%)	1 (3%)	21 (64%)		(70.8–98.8)	(58.7–99.7)	(76.1–100)	(51.5–97.9)
No	2 (6%)	10 (30%)	12 (36%)					
Vectorcardiogram (Anterior-Posterior)				**<** **.** **0001**	95%	91%	95%	91%
Yes	21 (64%)	1 (3%)	12 (67%)		(77.1–99.8)	(58.7–99.7)	(77.1–99.8)	(58.7–99.7)
No	1 (3%)	10 (30%)	21 (33%)					

CI, confidence interval; PPV, positive predictive value; NPV, negative predictive value; *T*-Test.

Bold values indicate statistical significance.

Patients with the T-wave plus-minus pattern had a significantly lower left ventricular ejection fraction (LVEF) value when compared to those without the pattern studied by ECG (34.3% ± 4.4% vs. 43.5% ± 8.0%; *p* = 0.002). A similar result was observed in the vectorcardiographic findings, which showed that patients with T-loop anterior-posterior morphology had significantly lower LVEF value compared to those without the pattern studied by VGC (34.3% ± 4.3% vs. 44.2% ± 7.9%; *p* = 0.002).

Patients with LVEF ≤ 40%, with sensitivity = 80%; specificity = 87%; positive predictive value = 95%; negative predictive value = 58% (*p* = 0.001), were identified by T-wave plus-minus pattern on the ECG In the VCG, the presence of the T-loop anterior-posterior pattern identified patients with LVEF ≤ 40%, with sensitivity = 84%; specificity = 87%; positive predictive value = 95%; negative predictive value = 64% (*p* < 0.001) (Table 2 and Supplementary Table S2, Panels C,D).

**Table 2:** Identification of patients with abnormal reduction of the left ventricular ejection fraction in moderate or severe degree, according to the presence of *plus-minus* (T-wave)/anterior-posterior (T-loop) in the test phase.

	Group		Total (*N* = 33)	*p*-value	Sensitivity	Specificity	PPV	NPV
	Left ventricular ejection fraction ≤40% (*N* = 25)	Left ventricular ejection fraction >40% (*N* = 8)			(CI 95%)	(CI 95%)	(CI 95%)	(CI 95%)
Electrocardiogram (*Plus-Minus*)				**0.0012**	80%	87%	95%	58%
Yes	20 (61%)	1 (3%)	21 (64%)		(59.3–93.1)	(47.3–99.6)	(76.1–99.8)	(27.6–84.8)
No	5 (15%)	7 (21%)	12 (36%)					
Vectorcardiogram (Anterior-Posterior)				**0.0005**	84%	87%	95%	64%
Yes	21 (64%)	1 (3%)	12 (67%)		(63.9–95.4)	(47.3–99.6)	(77.1–99.8)	(30.8–89.0)
No	4 (12%)	7 (21%)	21 (33%)					

CI, confidence interval; PPV, positive predictive value; NPV, negative predictive value; *T*-Test.

Bold values indicate statistical significance.

**Figure 5:**
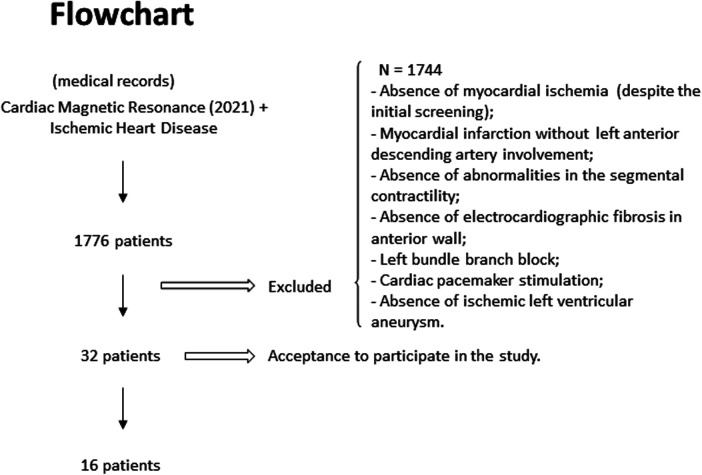
Flowchart of the participants’ selection in the validation phase.

In the validation phase, after the selection of 1,776 patients, only 35 presented eligibility criteria. Of these, we were able to contact only 19 patients. The excluded participants consisted of two patients who did not have a myocardial infarction area before the ECG, and another participant who had a left bundle branch block. Thus, 16 patients (aneurysm group - *n* = 8 and akinesia group - *n* = 8) concluded this phase (Figure 5).

Demographic variables were very alike in the test and validation phases. Mean age was 59.5 ± 8.0 years and 61.3 ± 8.6 years (*p* = 0.4) and male predominance (78.7% and 75.0%; *p* = 1.0) in both groups, respectively. The time interval between acute coronary syndrome and tests that defined left ventricle segmental contractility [echocardiogram with an ultrasonic enhancing agent for the test phase and cardiac magnetic resonance (CMR) for the validation phase] was 52.1 ± 41.8 months for the aneurysm group, and 56.6 ± 52.4 months for the akinesia group (*p* = 0.74). In the validation phase, the period between CMR and ECG, and VGC tests was 9.5 ± 3.6 months.

The validation phase showed that a plus-minus pattern in V2 and/or V3 and/or V4, identified patients with ventricular aneurysms. These results were similar to the test phase: sensitivity = 87%; specificity = 87%; positive predictive value = 87%; negative predictive value = 87%; accuracy = 87%, and likelihood ratio = 7 (*p* = 0.01). Same as occurred with the ECG findings, confirmation of the anterior-posterior vectorcardiographic pattern in V2 and/or V3 and/or V4 also identified patients with ventricular aneurysms, with results very similar to those of the test phase (sensitivity = 75%, specificity = 87%, positive predictive value = 87%, negative predictive value = 87%, accuracy = 81%, and likelihood ratio = 6; *p* = 0.04) (Table 3 and Supplementary Table S3, Panels E,F).

**Table 3:** Aneurysm group identification, according to the presence of *plus-minus* (T-wave)/anterior-posterior (T-loop) in the validation phase (segmental contractility analyzed by cardiac magnetic resonance).

	Group		Total (*N* = 16)	*p*-value	Sensitivity	Specificity	PPV	NPV
	Aneurysm (*N* = 8)	Akinesia (*N* = 8)			(CI 95%)	(CI 95%)	(CI 95%)	(CI 95%)
Electrocardiogram (*Plus-Minus*)				**0** **.** **01**	87%	87%	87%	87%
Yes	7 (44%)	1 (6%)	8 (50%)		(47.3–99.6)	(47.3–99.6)	(47.3–99.6)	(47.3–99.6)
No	1 (6%)	7 (44%)	8 (50%)					
Vectorcardiogram (Anterior-Posterior)				**0** **.** **04**	75%	87%	85%	77%
Yes	6 (38%)	1 (6%)	7 (44%)		(34.9–96.8)	(47.3–99.6)	(42.1–99.6)	(39.9–97.1)
No	2 (13%)	7 (44%)	9 (56%)					

CI, confidence interval; PPV, positive predictive value; NPV, negative predictive value; *T*-Test.

Bold values indicate statistical significance.

Agreement between the principal investigator and reviewer-1, for the electrocardiographic recognition of the T-wave plus-minus pattern in V2 and/or V3 and/or V4, was considered perfect, with *κ *=* *1.0 (*p* < 0.001), as shown in Supplementary Panel G.

Agreement among the principal investigator and reviewers 1 and 2 for vectorcardiographic recognition of T-loop anterior-posterior morphology in V2 and/or V3 and/or V4 was considered substantial (reviewer-1 - *κ* = 0.625, *p* = 0.012, and reviewer-2 - *κ* = 0.613, *p* = 0.013) (Supplementary, Panel H).

## 4. Discussion

In the United States of America, it is estimated that 4.9% of the population will be affected by acute myocardial infarction in 2,060 (25), resulting in around 800,000 people being diagnosed with a ventricular aneurysm. Nowadays, an estimated 1,400,000 people in the UK have survived an acute myocardial infarction, with up to 70,000 cases of ventricular aneurysm expected (26).

The electrocardiographic representation of ventricular aneurysms in ischemic patients has limited data. Initially, the analysis of the persistence of ST-segment elevation after acute myocardial infarction showed a sensitivity of up to 90% to identify ventricular aneurysm, however, when 2 mm ST-segment elevation is observed in conformity with Q-waves, sensitivity decreased to 60% (14). Despite the high sensitivity, this criterion does not confer specificity to the diagnosis of ventricular aneurysms. The residual ST-segment elevation after ST-segment elevation myocardial infarction predicts worse long-term clinical and structural deterioration at CMR (27).

The positive QRS complexes in aVR (15) are infrequently present in individuals with ventricular aneurysms, being a marker of high specificity, but low sensitivity (28). Finally, the results of the fragmentation of QRS complexes on the ECG in ischemic patients showed a sensitivity of 50% and specificity of 94.5% in identifying patients with ventricular aneurysms (16).

In the test phase, analysis of the studied population showed that the use of both parameters (presence of plus-minus T-wave pattern on ECG and anterior-posterior T-wave loop vectorcardiographic pattern) achieved an accuracy of 94% (*p* < 0.0001) in the identification of patients with an aneurysm. During the validation phase, the accuracy was up to 87% (*p* = 0.01), overcoming the data available in the literature so far.

It was also possible to infer that most of the patients with respectively the T-wave plus-minus, and the T-loop anterior-posterior aspects, present segmental involvement of the left ventricle with moderate or important systolic dysfunction (7). We noticed a 21.2% reduction in LVEF in the group with the electrocardiographic aspect studied (34.3% ± 4.4% vs. 43.5% ± 8.0%; *p* = 0.002); and in the VCG the LVEF reduction was 22.4% in those who had an anterior-posterior T-loop morphology (34.3% ± 4.3% vs. 44.2% ± 7.9%; *p* = 0.002).

The agreement among the reviewers' recognition of the T-wave plus-minus and anterior-posterior T-loop patterns in V2 and/or V3 and/or V4 was considered perfect and strong. This analysis proved to be extremely important for applying these methods in clinical practice.

### 4.1. Study strengths and limitations

This was a single center study. There was difficulty recruiting patients due to the pandemic moment and the cost involved in acquiring the echocardiographic enhancing agent contrast (SonoVue®). The population sample size limited the power for analysis of ventricular aneurysm recognition through the ECG and VCG and for the study of better agreement in identifying the T-wave plus-minus pattern and T-loop anterior-posterior morphology. Even though the evaluators were blinded to which group the ECG and VCG belonged to, they were aware of the object of the study (ventricular aneurysm).

Application of these methods in clinical practice may prove extremely reliable, since the accuracy obtained overcame the data available in the literature so far. Data suggest that it is possible to reproduce the electrovectorcardiographic findings by professionals with limited experience in analyzing electrocardiography and vectorcardiography methods.

## 5. Conclusion

Both the presence of the T-wave plus-minus ECG pattern, and the anterior-posterior expression of the T-wave loop in V2, and/or V3, and/or V4 enabled by the vectorcardiographic analysis, showed high accuracy for recognizing a left ventricular aneurysm in patients with ischemic heart disease.

## Data Availability

The original contributions presented in the study are included in the article/**Supplementary Material**, further inquiries can be directed to the corresponding author.
