# Supplementary material for: Electrovectorcardiographic study of left ventricular aneurysm in ischemic heart disease
Source: Front Cardiovasc Med. 2023 Dec 12;10:1275194. doi: 10.3389/fcvm.2023.1275194 (PMC10754535; doi:10.3389/fcvm.2023.1275194)
Supplement: Supplementary file 1 [file Table1.docx]

**SUPPLEMENTAL MATERIAL**

**Supplemental Table 1.** (Panels A and B).

**Panel A.** Analysis of the T-wave, T-loop and the left ventricular ejection fraction in the aneurysm group.

ECG = electrocardiogram; VCG = vectorcardiogram; LVEF (%) = left ventricular ejection fraction expressed as a percentage; 0 = absence of the T-wave *plus-minus* pattern in V2 and/or V3 and/or V4 on the electrocardiogram or absence of the anterior-posterior pattern of the T-loop in V2 and/or V3 and/or V4 in the vectorcardiogram; 1 = presence of the T-wave *plus-minus* pattern in V2 and/or V3 and/or V4 on the electrocardiogram or presence of the anterior-posterior pattern of the T-loop in V2 and/or V3 and/or V4 in the vectorcardiogram.

**Panel B.** Analysis of the T-wave, T-loop and the left ventricular ejection fraction in the akinesia group.

ECG = electrocardiogram; VCG = vectorcardiogram; LVEF (%) = left ventricular ejection fraction expressed as a percentage; 0 = absence of the T-wave *plus-minus* pattern in V2 and/or V3 and/or V4 on the electrocardiogram or absence of the anterior-posterior pattern of the T-loop in V2 and/or V3 and/or V4 in the vectorcardiogram; 1 = presence of the T-wave *plus-minus* pattern in V2 and/or V3 and/or V4 on the electrocardiogram or presence of the anterior-posterior pattern of the T-loop in V2 and/or V3 and/or V4 in the vectorcardiogram.

**Supplemental Table 2**. (Panels C and D).

**Panel C.** Relationship: left ventricular ejection fraction and presence of the T-wave *plus-minus* on the electrocardiogram.

LVEF (%) = left ventricular ejection fraction expressed as a percentage; LVEF (%) and *plus-minus* existent = presence of the T-wave *plus-minus* pattern in V2 and/or V3 and/or V4 on the electrocardiogram and left ventricular ejection fraction of the analysed patient. LVEF (%) and *plus-minus* absent = absence of the T-wave *plus-minus* pattern in V2 and/or V3 and/or V4 on the electrocardiogram and left ventricular ejection fraction of the analysed patient.

**Panel D.** Relationship: left ventricular ejection fraction and presence of the anterior-posterior T-loop on the vectorcardiogram.

LVEF (%) = left ventricular ejection fraction expressed as a percentage; LVEF (%) and anterior-posterior existent = presence of the anterior-posterior T-loop pattern in V2 and/or V3 and/or V4 on the vectorcardiogram and left ventricular ejection fraction of the analysed patient. LVEF (%) and anterior-posterior absent = absence of the anterior-posterior T-loop pattern in V2 and/or V3 and/or V4 on the vectorcardiogram and left ventricular ejection fraction of the analysed patient.

**Supplemental Table 3.** (Panels E to G).

**Panel E.** T-wave analysis of the aneurysm group in the validation phase.

ECG = electrocardiogram; VCG = vectorcardiogram; 0 = absence of the T-wave *plus-minus* pattern in V2 and/or V3 and/or V4 on the electrocardiogram or absence of the anterior-posterior pattern of the T-loop in V2 and/or V3 and/or V4 in the vectorcardiogram; 1 = presence of the T-wave *plus-minus* pattern in V2 and/or V3 and/or V4 on the electrocardiogram or presence of the anterior-posterior pattern of the T-loop in V2 and/or V3 and/or V4 in the vectorcardiogram.

**Panel F.** T-wave analysis of the akinesia group in the validation phase.

ECG = electrocardiogram; VCG = vectorcardiogram; 0 = absence of the T-wave *plus-minus* pattern in V2 and/or V3 and/or V4 on the electrocardiogram or absence of the anterior-posterior pattern of the T-loop in V2 and/or V3 and/or V4 in the vectorcardiogram; 1 = presence of the T-wave *plus-minus* pattern in V2 and/or V3 and/or V4 on the electrocardiogram or presence of the anterior-posterior pattern of the T-loop in V2 and/or V3 and/or V4 in the vectorcardiogram.

**Panel G.** Classification of the presence or absence of the T wave *plus-minus* pattern in the validation phase, according to reviewer-1.

ECG = electrocardiogram; 0 = absence of the T-wave *plus-minus* pattern in V2 and/or V3 and/or V4 on the electrocardiogram; 1 = presence of the T-wave *plus-minus* pattern in V2 and/or V3 and/or V4 on the electrocardiogram.

**Panel H.** Classification of the presence or absence of the anterior-posterior pattern of the T-loop in the vectorcardiogram in the validation phase, according to the reviewers.

VCG = vectorcardiogram; 0 = absence of the anterior-posterior pattern of the T-loop in V2 and/or V3 and/or V4 in the vectorcardiogram; 1 = presence of the anterior-posterior pattern of the T-loop in V2 and/or V3 and/or V4 in the vectorcardiogram.
